# Species of associated bryophytes and their effects on the yield and quality of *Dendrobium nobile*

**DOI:** 10.1186/s12870-023-04503-5

**Published:** 2023-10-26

**Authors:** Mingsong Li, Jinling Li, Lujun Deng, Zhi Zhao, Chunli Luo, Fulai Luo, Hualei Wang, Jiyong Yang

**Affiliations:** 1 College of Agriculture, Guizhou University, Guiyang, 550025 China; 2 Key Laboratory of Breeding and Cultivation of Medicinal Plants of Guizhou Province, Guiyang, 550025 China; 3 Institute of Biotechnology, Guizhou Provincial Academy of Agricultural Sciences, Guiyang, 550006 China; 4 Chishui Xintian Traditional Chinese Medicine Industry Development Co. Ltd, Chishui, 64700 China

**Keywords:** *Dendrobium nobile*, Bryophyte, Associative relationships, Yield and quality

## Abstract

**Background:**

*Dendrobium nobile* has unique growth environment requirements, and unstable yields and high management costs are the key factors restricting the development of its imitation wild cultivation industry. The present study explored the effects of different associated bryophyte species on the yield and quality of *D. nobile* to clarify the dominant bryophyte species associated with *D. nobile* and to provide a scientific basis for the rational cultivation and quality evaluation of *D. nobile*.

**Results:**

The growth of *D. nobile* was closely related to the microenvironment of the Danxia stone, and the different associated bryophytes had different effects on *D. nobile* growth. There was a rich variety of bryophytes associated with *D. nobile*, with a total of 15 families, 24 genera and 31 species of bryophytes identified in the study area, including 13 families, 22 genera and 29 species of mosses and 2 families, 2 genera and 2 species of liverworts, and mosses predominated in the association with *D. nobile*. Usually, 3–9 species of bryophytes were growing in association with *D. nobile*, among which associations of 5–6 bryophytes species were more common, and the bryophytes associated with *D. nobile* were only related to the species to which they belonged. The dry matter accumulation, quality and mineral content of *D. nobile* differed significantly among different bryophyte species. The coefficients of variation of dry matter accumulation, dendrobine content and content of 11 mineral elements of *D. nobile* in the 35 sample quadrats were 25.00%, 21.08%, and 11.33–57.96%, respectively. The biomass, dendrobine content and mineral content of *D. nobile* were analysed by principal component analysis (PCA) and membership function. The results showed that each evaluation method initially screened *Trachycystis microphylla* and *Leucobryum juniperoideum* as the dominant associated bryophytes in the preliminary identification analysis, and the frequency of occurrence and coverage of the two bryophytes were significantly higher than those of the remaining bryophytes. It was determined that *T. microphylla* and *L. juniperoideum* were the dominant associated bryophytes.

**Conclusions:**

There is a rich variety of bryophytes associated with *D. nobile*. The yield and quality of *D. nobile* differed significantly among different bryophyte species. *T. microphylla* and *L. juniperoideum* were the dominant associated bryophytes, and were the two bryophytes associated with *D. nobile* through mixed growth.

## Background

*Dendrobium nobile* is a perennial epiphytic herb in the family Orchidaceae whose dried stems are used as medicine [1]. It is a rare and valuable Chinese medicinal herb under secondary protection in China. *D. nobile* is rich in polysaccharides, alkaloids and other active substances, and it is used for improving immunity, lowering blood sugar, and as an antitumour agent, among other uses, and its medicinal value is very high [2, 3]. *D. nobile* has a long history of artificial cultivation in Chishui City, Guizhou Province, China. Because of its unique geological features and high-temperature and high-humidity climate, Chishui City gradually became the main source of raw herbs for the *D. nobile* market. *D. nobile* has been grown in Chishui City under the imitation wild cultivation mode since the early 1980s to pilot artificial cultivation. In 1998, the State Administration of Traditional Chinese Medicine (State Administration of Traditional Chinese Medicine Sheng [1998] No. 17 document) approved the establishment of a *D. nobile* production base in Chishui City to develop the original *D. nobile* ecological planting, which is China's only national *D. nobile* base. In 2006, *D. nobile* from Chishui was awarded the designation of national geographical indication product, and in 2019, it was selected for China's Agricultural Brand Catalogue. At present, the original area of *D. nobile* cultivation in Chishui on Danxia stone under forest is 6,432 hectares, accounting for more than 90% of the national *D. nobile* cultivation area and product quantity.

*D. nobile* has strict growth requirements, preferring high-temperature, high-humidity, cool environments, and it can be grown at an altitude of 300–1,000 m. It often grows attached to rocks or tree trunks with humus and bryophyte aggregates. The terrain where it grows is mostly cliffs, usually shaded by trees and near water sources, and the plants require sufficient oblique sunlight and grow slowly [4–6]. For *D. nobile* planted on Danxia stone under imitation wild cultivation, planting to harvesting generally takes over 3 years and requires substantial financial investment in labour and materials, and although the management cycle is long, the yield is unstable. This is the key factor restricting the development of the *D. nobile* industry at present. Our preliminary research found that the growth of *D. nobile* planted on Danxia stone under imitation wild cultivation was closely related to its microenvironment. The different shade, direction and surrounding water environment conditions of each Danxia stone scattered in the planting area led to differences in light, temperature, water and fertiliser use, and bryophyte species on the surface layer. Additionally, there were large differences in the growth and biomass of *D. nobile* and associated bryophyte species in the different microenvironments. Therefore, we hypothesised that there are differences in the degree of association between different bryophytes and *D. nobile*. According to the results of previous studies, bryophytes have different morphological and physiological characteristics [7–9] that have distinct effects on plant growth. The effect of bryophytes on plant growth may depend on a variety of external factors, and bryophytes can indirectly affect plants positively or negatively by regulating water retention capacity [10, 11], reducing temperature fluctuations [7, 9], increasing the rate of apoplastic decomposition to provide nutrients [12, 13], and potentially contributing to soil nitrogen levels through symbiotic relationships with nitrogen-fixing cyanobacteria [14, 15].

To date, studies on the factors influencing the yield and quality of *D. nobile* have focused on factors such as harvesting period [4, 16], cultivation substrate [17–19], and climatic conditions [20–22]. However, the critical role of the microenvironment in the growth of *D. nobile* has been neglected, and investigations of the *D. nobile* microenvironment, associated bryophyte species, and the effect of bryophytes on the yield and quality of *D. nobile* have not yet been reported. Therefore, this study was conducted to identify the bryophyte species associated with *D. nobile* through field survey sampling, analyse the effects of different bryophytes on the yield and quality of *D. nobile*, and clarify the dominant bryophytes associated with *D. nobile*. Through investigation of the formation of Danxia stone microenvironment and its interactions with *D. nobile* and associated bryophytes, we aim to provide a reference for the rational cultivation and quality evaluation of *D. nobile*.

## Results and analyses

### Survey of associated bryophyte species in the study area

**Fig. 1:**
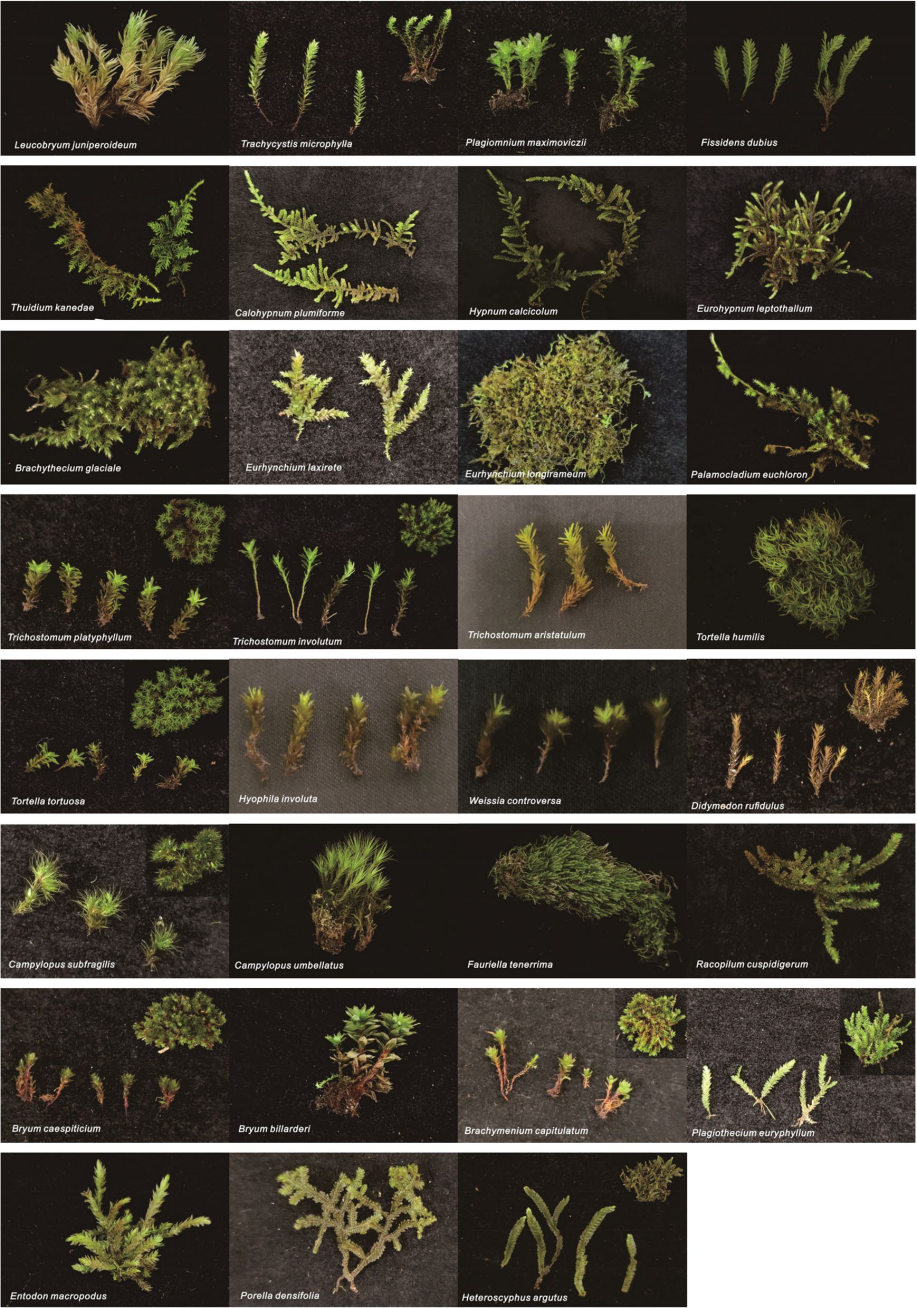
Representative specimens of the 31 bryophyte species associated with *Dendrobium* *nobile*

The bryophytes associated with *D. nobile* were identified as 31 species in 24 genera and 15 families (Fig. 1), and the species composition of the samples is shown in Table 1. As shown in the table, among the bryophytes associated with *D. nobile*, there were 29 species in 22 genera and 13 families of mosses and 2 species in 2 genera and 2 families of liverworts. The proportions of mosses in the total families, genera, and species reached 86.67%, 91.67%, and 93.55%, respectively, showing that mosses associated with *D. nobile* are better adapted to live in the local lithophytic environment than liverworts.

**Table 1:** Species composition of 31 bryophytes associated with *Dendrobium nobile*

Species	Genus	Family
*Leucobryum juniperoideum*	*Leucobryum*	Leucobryaceae
*Trachycystis microphylla*	*Trachycystis*	Mniaceae
*Plagiomnium maximoviczii*	*Plagiomnium*	Mniaceae
*Fissidens dubius*	*Fissidens*	Fissidentaceae
*Thuidium kanedae*	*Thuidium*	Thuidiaceae
*Calohypnum plumiforme*	*Hypnum*	Hypnaceae
*Hypnum calcicolum*	*Hypnum*	Hypnaceae
*Eurohypnum leptothallum*	*Eurohypnum*	Hypnaceae
*Brachythecium glaciale*	*Brachythecium*	Brachytheciaceae
*Eurhynchium laxirete*	*Eurhynchium*	Brachytheciaceae
*Eurhynchium longirameum*	*Eurhynchium*	Brachytheciaceae
*Palamocladium euchloron*	*Palamocladium*	Brachytheciaceae
*Trichostomum platyphyllum*	*Trichostomum*	Pottiaceae
*Trichostomum involutum*	*Trichostomum*	Pottiaceae
*Trichostomum aristatulum*	*Trichostomum*	Pottiaceae
*Tortella humilis*	*Tortella*	Pottiaceae
*Tortella tortuosa*	*Tortella*	Pottiaceae
*Hyophila involuta*	*Hyophila*	Pottiaceae
*Weissia controversa*	*Weissia*	Pottiaceae
*Didymodon rufidulus*	*Didymodon*	Pottiaceae
*Campylopus subfragilis*	*Campylopus*	Dicranaceae
*Campylopus umbellatus*	*Campylopus*	Dicranaceae
*Fauriella tenerrima*	*Fauriella*	Theliaceae
*Racopilum cuspidigerum*	*Racopilum*	Racopilaceae
*Bryum caespiticium*	*Bryum*	Bryaceae
*Bryum billarderi*	*Bryum*	Bryaceae
*Brachymenium capitulatum*	*Brachymenium*	Bryaceae
*Plagiothecium euryphyllum*	*Plagiothecium*	Plagiotheciaceae
*Entodon macropodus*	*Entodon*	Entodontaceae
*Porella densifolia*	*Porella*	Porellaceae
*Heteroscyphus argutus*	*Heteroscyphus*	Geocalycaceae

Analysing the composition of the 31 bryophyte families (Table 2), the only family containing five genera was Bryophyta. There were one and three families containing three and two genera, respectively, with nine species of plants, accounting for 26.67% of the total number of families. The remaining families all contained only one genus, accounting for 66.67% of the total number of families, indicating a high degree of richness, although there are few species of bryophytes associated with *D. nobile* in the region.

**Table 2:** Composition of 31 bryophyte families

Number of genera included in a single family	Number of families	Proportion of total families/%	Number of genera	Proportion of total genera/%	Number of species	Proportion of total number of species/%
5	1	6.25	5	20.83	8	25.81
3	1	6.25	3	12.50	4	12.90
2	3	18.75	6	25.00	5	16.13
1	11	68.75	11	45.83	14	45.16
Total	16	100.00	23	100.00	31	100.00

Analysing the composition of the 31 bryophyte genera (Table 3), there was one genus containing three species of plants, five genera containing two species of plants, and 18 genera containing one species of plants, which accounted for 75.00% of the total number of genera and 58.06% of the total number of species. This indicates that the monotypic bryophyte composition is higher in this region and that the degree of species diversity of bryophytes is high.

**Table 3:** Composition of 31 bryophyte genera

Number of species in a single genus	Number of genera	Proportion of total genera/%	Number of species	Proportion of total species/%
3	1	4.17	3	9.68
2	5	20.83	10	32.26
1	18	75.00	18	58.06
Total	24	100.00	31	100.00

### Analysis of associated bryophyte species on 35 Danxia stones

**Fig. 2:**
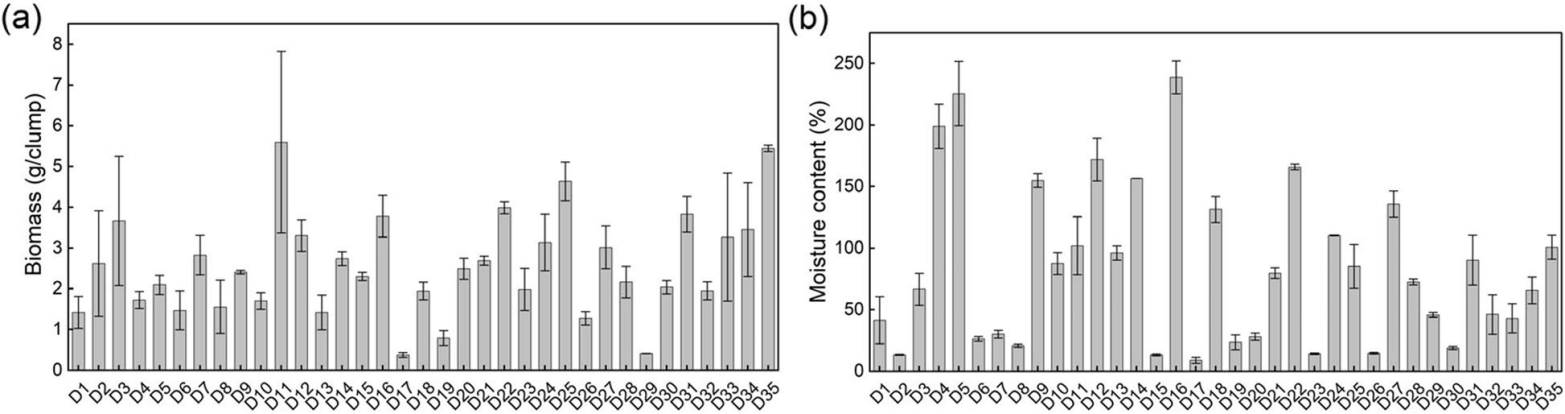
Biomass accumulation (**a**) and moisture content (**b**) of bryophytes in 35 quadrats on Danxia stone

**Fig. 3:**
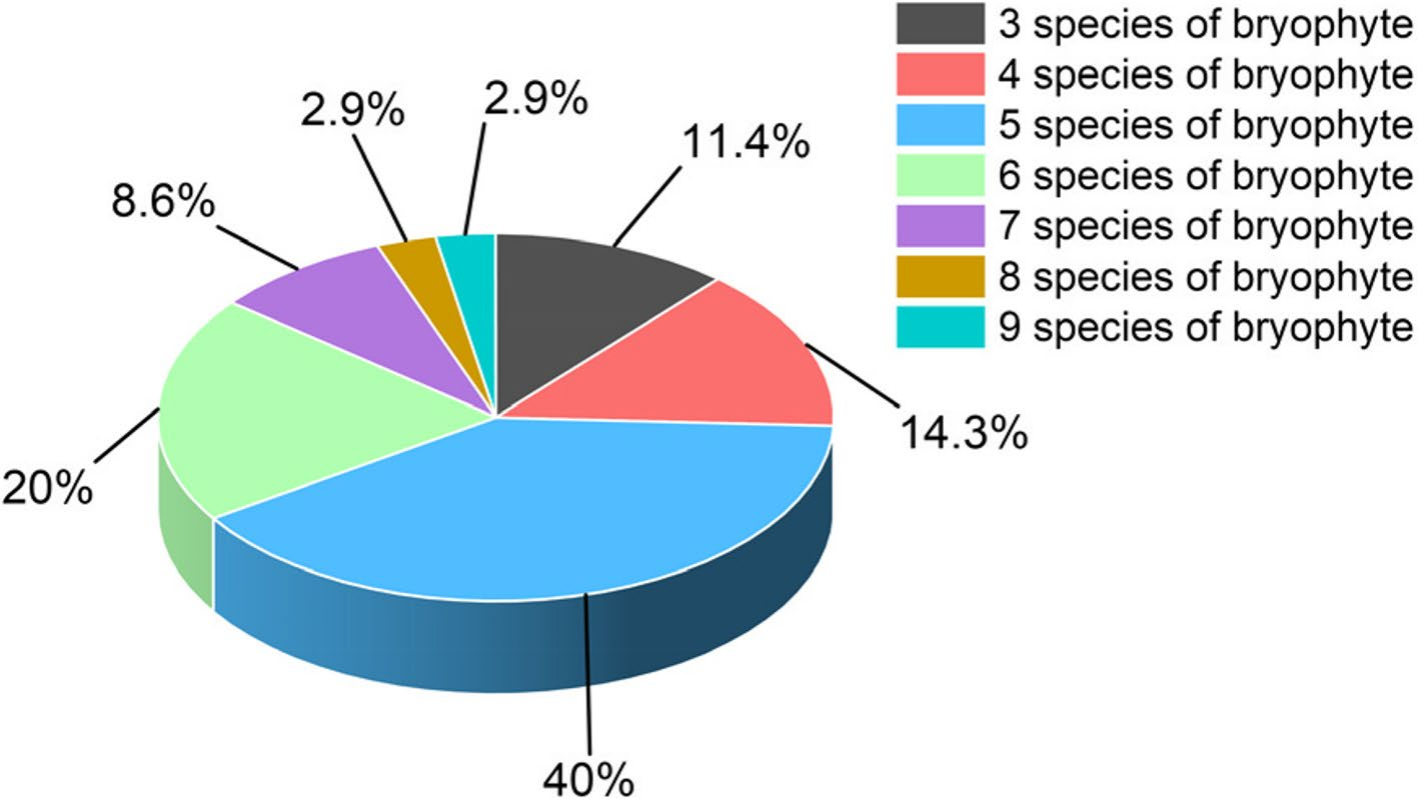
Differences in bryophyte species occurrence in 35 quadrats on Danxia stone

The species, cover, moisture content and biomass of bryophytes were determined for 35 Danxia stones (Table 4 and Fig. 2). According to Table 4, a total of 28 bryophyte species were obtained from the 35 quadrats, and only three bryophytes were missing compared to the bryophyte species collected during the previous ecological surveys in the study area, suggesting that the 35 Danxia stones are representative of the distribution of associated bryophytes in the study area. Three bryophytes, *Eurhynchium laxirete*, *Hypnum calcicolum*, and *Porella densifolia*, were missing from the 35 quadrats, suggesting that these three bryophytes have a relatively weak associative relationship with *D. nobile*. According to the results of the survey, the bryophytes associated with *D. nobile* were all a mixture of multiple bryophytes, and no single species of bryophyte was found. There were considerable differences in bryophyte species between different Danxia stones, with 3–9 species of bryophyte epiphytic on one Danxia stone. The attachment of 5–6 bryophyte species to one Danxia stone was more common (Fig. 3), accounting for 60.00% of the total number of quadrats, followed by the attachment of 3, 4, and 7 bryophyte species, accounting for 34.29%, and the attachment of 8–9 bryophyte species, accounting for only 5.71%. In addition, bryophytes in 35 quadrats showed large differences in moisture content and biomass accumulation (Fig. 2). The moisture content of bryophytes in the 35 quadrats ranged from 8.81–238.78%, with a coefficient of variation of 76.27%, and the bryophyte biomass ranged from 0.38–5.60 g/clump, with a coefficient of variation of 48.05%.

**Table 4:** Bryophyte species and their cover in 35 quadrats on Danxia stone

Number	Bryophyte species	Cover/%	Number	Bryophyte species	Cover/%	Number	Bryophyte species	Cover/%
D1	*B. glaciale*	5	D13	*L. juniperoideum*	10	D24	*T. microphylla*	90
	*B. caespiticium*	5		*C. umbellatus*	5		*L. juniperoideum*	20
	*L. juniperoideum*	5		*B. glaciale*	5		*B. caespiticium*	5
	*F. tenerrima*	5		*C. plumiforme*	5		*C. subfragilis*	1
	*T. microphylla*	50		*P. euryphyllum*	5		*H. argutus*	1
D2	*C. umbellatus*	1		*T. kanedae*	1	D25	*T. tortuosa*	80
	*E. leptothallum*	90	D14	*L. juniperoideum*	50		*R. cuspidigerum*	50
	*B. capitulatum*	5		*T. microphylla*	50		*F. dubius*	50
D3	*F. dubius*	5		*P. euryphyllum*	5		*E. longirameum*	30
	*B. billarderi*	1		*R. cuspidigerum*	5		*B. billarderi*	1
	*E. leptothallum*	90	D15	*T. kanedae*	1		*T. kanedae*	1
	*F. tenerrima*	1		*R. cuspidigerum*	50	D26	*T. microphylla*	90
	*B. glaciale*	10		*T. kanedae*	10		*T. tortuosa*	30
	*T. kanedae*	1		*B. glaciale*	5		*B. caespiticium*	10
	*T. microphylla*	10		*F. dubius*	5		*L. juniperoideum*	5
	*L. juniperoideum*	1		*E. macropodus*	1		*P. euchloron*	5
D4	*H. argutus*	20	D16	*L. juniperoideum*	60		*R. cuspidigerum*	1
	*R. cuspidigerum*	10		*T. microphylla*	30	D27	*T. microphylla*	90
	*T. involutum*	50		*F. dubius*	5		*T. tortuosa*	10
	*T. microphylla*	5		*H. argutus*	5		*B. billarderi*	10
D5	*B. billarderi*	5		*C. subfragilis*	5		*B. glaciale*	10
	*L. juniperoideum*	20		*R. cuspidigerum*	5		*R. cuspidigerum*	5
	*B. billarderi*	1		*T. kanedae*	1		*L. juniperoideum*	1
	*T. microphylla*	50	D17	*B. glaciale*	50	D28	*R. cuspidigerum*	70
	*L. juniperoideum*	50		*B. caespiticium*	20		*B. caespiticium*	60
D6	*R. cuspidigerum*	10		*T. kanedae*	1		*H. involuta*	50
	*B. capitulatum*	80		*C. umbellatus*	1		*F. dubius*	5
	*T. kanedae*	30		*L. juniperoideum*	1		*B. billarderi*	1
	*C. plumiforme*	5	D18	*T. microphylla*	80		*W. controversa*	1
	*B. glaciale*	1		*C. umbellatus*	5	D29	*B. caespiticium*	50
D7	*T. microphylla*	70		*L. juniperoideum*	5		*T. microphylla*	50
	*T. kanedae*	1		*T. platyphyllum*	5		*L. juniperoideum*	10
	*F. dubius*	5		*D. rufidulus*	5		*F. tenerrima*	5
	*E. leptothallum*	5	D19	*L. juniperoideum*	10	D30	*R. cuspidigerum*	90
	*T. aristatulum*	1		*T. platyphyllum*	10		*T. tortuosa*	50
D8	*B. caespiticium*	5		*F. dubius*	5		*L. juniperoideum*	10
	*C. plumiforme*	1		*T. microphylla*	5	D31	*T. microphylla*	80
	*T. kanedae*	1		*R. cuspidigerum*	1		*T. kanedae*	30
	*R. cuspidigerum*	10	D20	*T. microphylla*	70		*B. billarderi*	10
	*B. glaciale*	5		*L. juniperoideum*	10		*C. umbellatus*	5
D9	*T. microphylla*	95		*F. tenerrima*	10	D32	*B. glaciale*	80
	*E. leptothallum*	5		*C. plumiforme*	1		*T. microphylla*	50
	*C. plumiforme*	1		*T. kanedae*	1		*F. tenerrima*	10
	*L. juniperoideum*	5	D21	*T. microphylla*	90		*T. tortuosa*	10
D10	*T. microphylla*	90		*L. juniperoideum*	10		*C. umbellatus*	50
	*F. dubius*	5		*B. caespiticium*	10	D33	*T. microphylla*	90
	*T. tortuosa*	3		*T. tortuosa*	1		*T. platyphyllum*	5
	*F. tenerrima*	2	D22	*T. microphylla*	90		*T. tortuosa*	5
	*L. juniperoideum*	1		*L. juniperoideum*	10		*L. juniperoideum*	5
D11	*T. microphylla*	90		*T. tortuosa*	5	D34	*R. cuspidigerum*	90
	*L. juniperoideum*	1		*P. maximoviczii*	5		*B. caespiticium*	10
	*H. argutus*	1		*F. dubius*	5		*B. billarderi*	5
D12	*T. microphylla*	90		*B. billarderi*	1		*W. controversa*	10
	*B. glaciale*	1		*R. cuspidigerum*	1		*E. leptothallum*	5
	*C. umbellatus*	1		*T. platyphyllum*	1		*T. involutum*	70
	*L. juniperoideum*	1		*B. capitulatum*	1	D35	*C. subfragilis*	50
	*C. plumiforme*	1	D23	*T. microphylla*	80		*B. billarderi*	50
	*H. argutus*	1		*T. tortuosa*	50		*F. dubius*	5
	*B. billarderi*	1		*B. caespiticium*	10		*L. juniperoideum*	1
				*R. cuspidigerum*	10		*H. argutus*	1
				*L. juniperoideum*	1		*T. tortuosa*	1
				*T. humilis*	1			

Since each bryophyte grows mixed within the same quadrat, the sum of coverage is not 100%

### Frequency of occurrence and coverage of different bryophytes

**Fig. 4:**
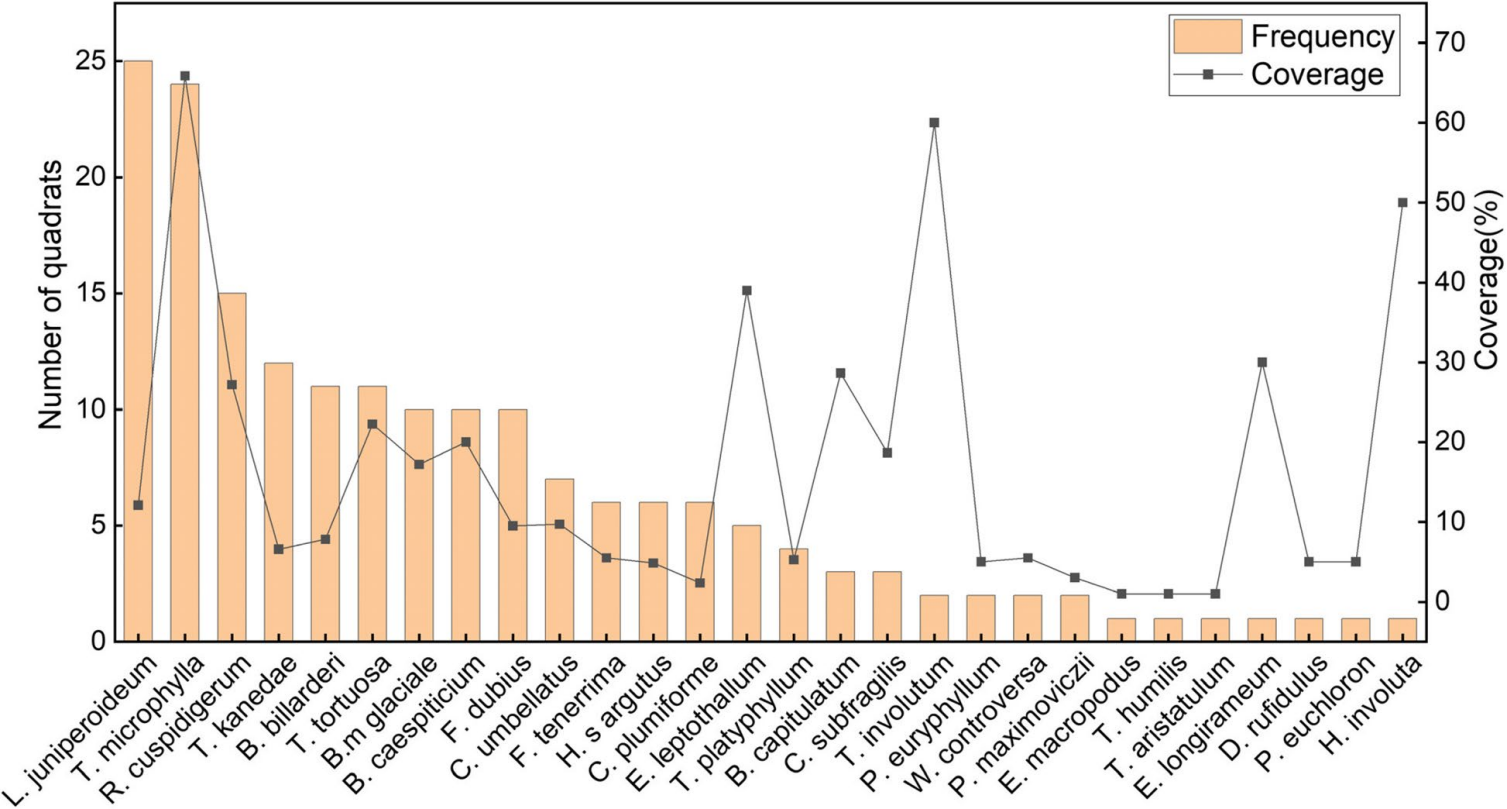
Frequency and coverage of 28 species of bryophytes

The frequency and coverage of different species of bryophytes in the 35 quadrats were determine (Fig. 4). There were large differences in the frequency of different species of bryophytes. Among them, *Leucobryum juniperoideum* and *Trachycystis microphylla* were the most common, with both bryophytes growing on 70% of the quadrat at frequencies of 71.43% and 68.57%, respectively. The frequency of 7 bryophytes, *Racopilum cuspidigerum*, *Thuidium kanedae*, *Bryum billarderi*, *Tortella tortuosa*, *Brachythecium glaciale*, *Bryum caespiticium*, and *Fissidens dubius*, ranged between 28.57–42.86%. The frequency of 14 bryophytes, *Trichostomum platyphyllum*, *Brachymenium capitulatum*, *Campylopus subfragilis*, *Trichostomum involutum*, *Plagiothecium euryphyllum*, *Weissia controversa*, *Plagiomnium maximoviczii*, *Entodon macropodus*, *Tortella humilis*, *Trichostomum aristatulum*, *Eurhynchium longirameum*, *Didymodon rufidulus*, *Palamocladium euchloron*, *and Hyophila involuta*, was the lowest, ranging from 2.86–11.43%. Additionally, the coverage of different species of bryophytes varied considerably, ranging from 1.00–65.87%. Three bryophytes, *T. microphylla*, *T. involutum*, and *H. involuta*, showed large coverage, at more than 50%. The coverage of 6 bryophytes, *Eurohypnum leptothallum*, *E. longirameum*, *B. capitulatum*, *R. cuspidigerum*, *T. tortuosa*, and *B. caespiticium*, was the next highest, ranging from 20.00–39.00%. The coverage of 3 bryophytes, *E. macropodus*, *T. humilis* and *T. aristatulum*, was the smallest, at 1.00%.

**Fig. 5:**
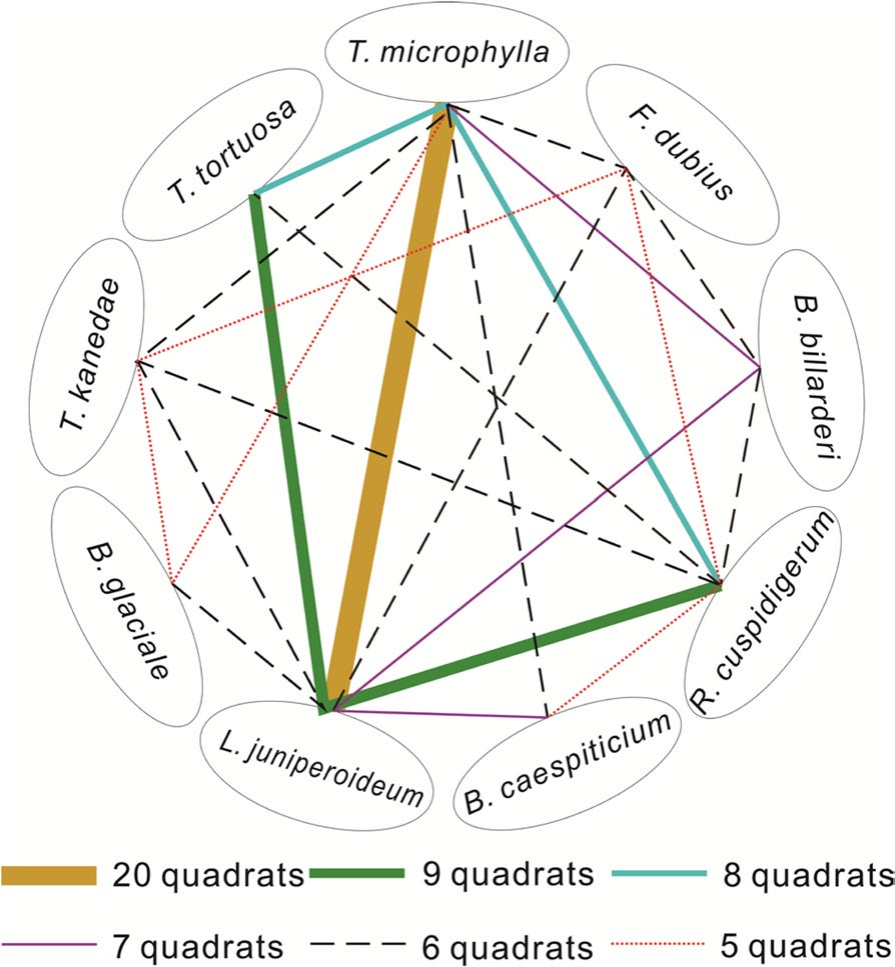
Number of quadrats in which 9 bryophyte species pairs were found growing in association

To clarify the associations among different bryophytes species, we determined the number of quadrats of all species of bryophytes growing mixed with each other and classified the two bryophytes species showing mixed growth in greater than 5 quadrats as preliminarily identified dominant associated bryophytes (Fig. 5). The results showed that only 9 species of bryophytes, *L. juniperoideum*, *T. microphylla*, *R. cuspidigerum*, *T. kanedae*, *B. billarderi*, *T. tortuosa*, *B. glaciale*, *B. caespiticium*, and *F. dubius*, grow in associations containing more than 5 species. Among them, *L. juniperoideum* had the highest frequency of mixed growth with *T. microphylla* (20 times), which was significantly higher than that of the remaining bryophytes. The frequency of mixed growth among the bryophytes was mostly 6 times, with a total of 9 pairs of bryophytes.

We compared the frequency of each bryophyte with its coverage and found a weak correlation. Only 9 bryophyte species, *T. microphylla*, *P. euryphyllum*, *W. controversa*, *P. maximoviczii*, *E. macropodus*, *T. humilis*, *T. aristatulum*, *D. rufidulus*, and *P. euchloron*, showed a high correlation between frequency and coverage. In addition, the frequency and coverage of each bryophyte was compared with its family/genus, and it was found that the frequency and coverage of bryophytes were not strongly correlated with their family/genus. This suggests that the bryophytes associated with *D. nobile* are related only to the species to which they belong.

### Effect of different bryophyte species on the biomass of *D. nobile*

**Fig. 6:**
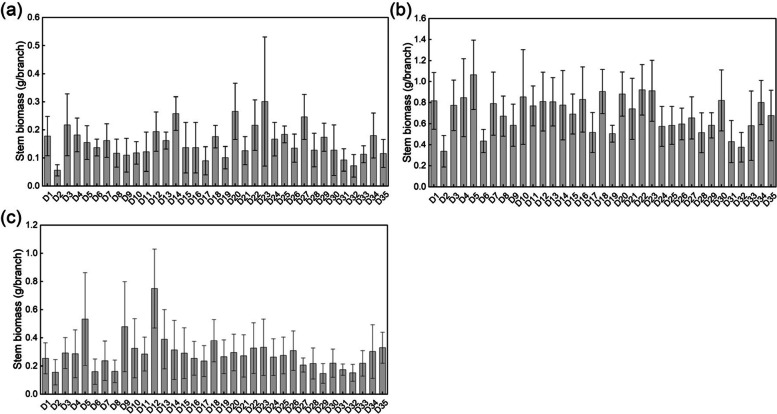
Dry matter accumulation of 1-year-old (**a**), 2-year-old (**b**), and 3-year-old (**c**) D. nobile stems from 35 quadrats

There were differences in the dry matter accumulation of *D. nobile* stems associated with different bryophyte species (Fig. 6). The dry matter accumulation of 1-, 2- and 3-year-old stems from 35 quadrats was 0.056–0.301 g/branch, 0.338–1.065 g/branch, and 0.147–0.75 g/branch, with coefficients of variation of 35.98%, 24.50%, and 40.13%, respectively.

### Effect of different species of bryophytes on the quality of *D. nobile*

**Fig. 7:**
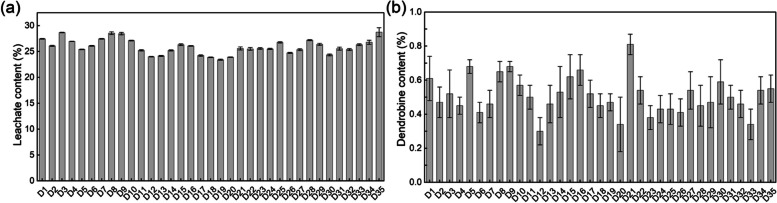
Leachate (**a**) and dendrobine (**b**) content of 3-year-old D. nobile stems from 35 quadrats

The harvesting of *D. nobile* is usually performed by selecting stems older than 3 years. Therefore, in this experiment, we only determined the quality and mineral content of the 3-year-old stems. The leachate and dendrobine content of the 3-year-old stems of *D. nobile* from 35 quadrats are shown in Fig. 7. The leachate content ranged from 23.41–28.74%, with little variation in content and a coefficient of variation of 5.46%. In contrast, the dendrobine content significantly differed, ranging from 0.30–0.81%, with a coefficient of variation of 21.08%.

### Effect of different species of bryophytes on the mineral element content of *D. nobile*

**Fig. 8:**
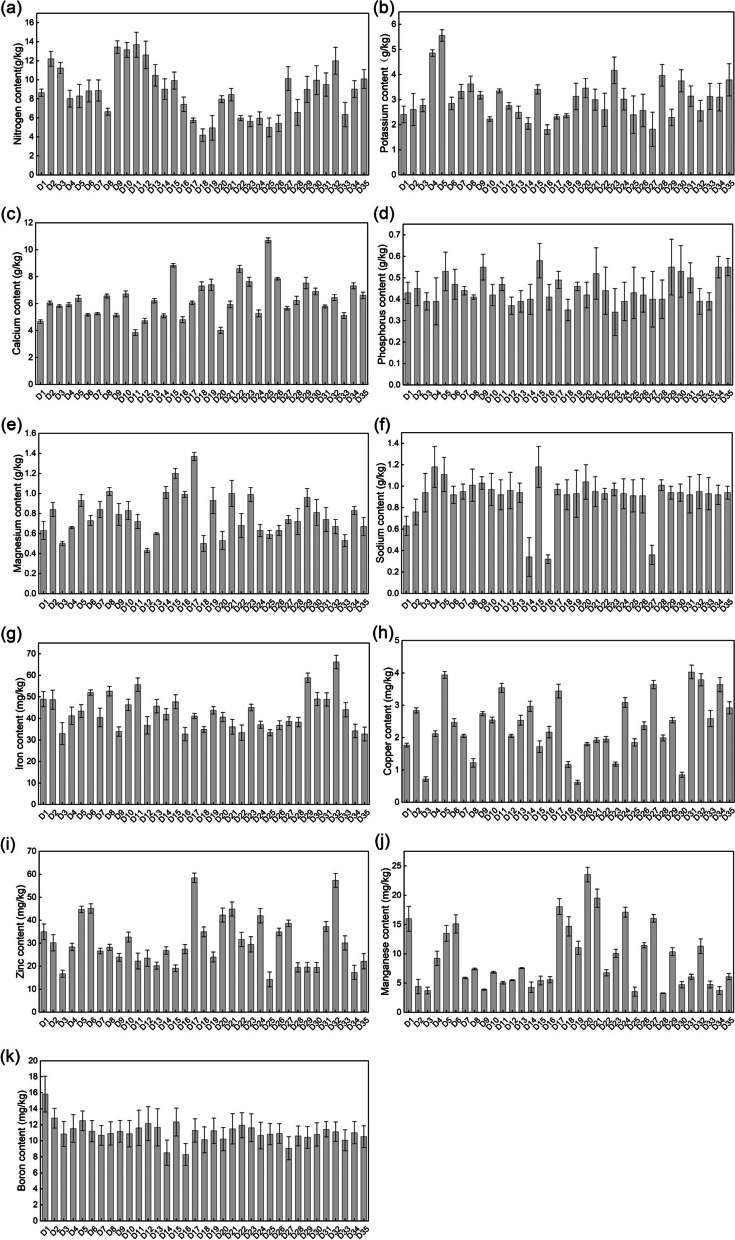
Content of 11 mineral elements in 3-year-old stems of D. nobile from 35 quadrats

The content of 11 mineral elements in the 3-year-old stems of *D. nobile* varied among the 35 quadrats (Fig. 8). The contents of the 11 elements, N, K, Ca, P, Mg, Na, Fe, Cu, Zn, Mn, and B, were 4.18–13.69 g·kg^−1^, 1.81–5.56 g·kg^−1^, 3.85–10.70 g·kg^−1^, 0.34–0.58 g·kg^−1^, 0.43–1.37 g·kg^−1^, 0.32–1.18 g·kg^−1^, 32.75–66.18 mg·kg^−1^, 0.62–4.03 mg·kg^−1^, 14.27–58.44 mg·kg^−1^, 3.26–23.54 mg·kg^−1^, 8.29–15.83 mg·kg^−1^, respectively. The content of the elements followed the order Mn > Cu > Zn > N > K > Mg > Ca > Na > Fe > P > B, with coefficients of variation ranging from 11.33–57.96%.

### Correlation analysis

**Fig. 9:**
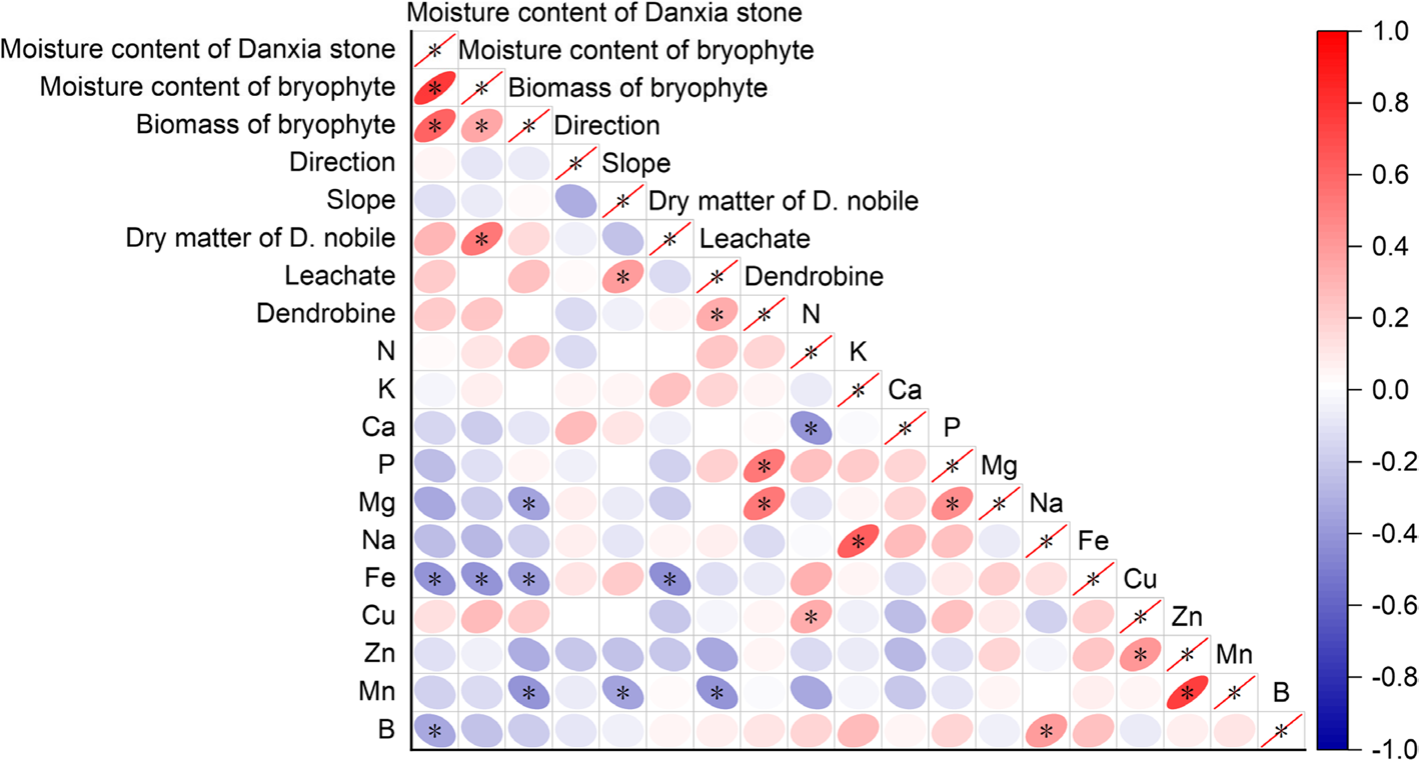
Correlation analysis of environmental factors with dry matter, quality and mineral element content of *D.* *nobile*

The results of the correlation analysis between environmental factors and dry matter, quality and mineral element content of *D. nobile* are shown in Fig. 9. The results of the correlation analyses of five environmental factors—direction, slope and moisture content of Danxia stone and moisture content and biomass of bryophytes—showed that the moisture content of Danxia stone was significantly and positively correlated with the moisture content and biomass of bryophytes (*P* < 0.05), but the correlations among the remaining environmental factors were not significant.

Correlation analyses of dry matter accumulation, quality and mineral content of *D. nobile* associated with different species of bryophytes showed that among 14 indicators, 13 pairs of traits were significantly or highly significantly correlated. Dry matter showed a significant negative correlation (*P* < 0.05) only with elemental Fe. Leachate showed a significant positive correlation (*P* < 0.05) with dendrobine and a significant negative correlation (*P* < 0.05) with Mn. Dendrobine was significantly and positively correlated (*P* < 0.05) with P and Mg. There were 7 pairs of traits that were significantly or highly significantly correlated among the 11 mineral elements. Six pairs of elements, N-Cu, K-Na, P-Mg, Mg-B, Cu–Zn, and Zn-Mn, were significantly positively correlated (*P* < 0.05), while N-Ca was significantly negatively correlated (*P* < 0.05).

The results of the correlation analysis between environmental factors and the dry matter accumulation, quality and mineral element content of *D. nobile* showed that the moisture content of Danxia stone was significantly negatively correlated with Fe and B (*P* < 0.05); the moisture content of bryophytes was significantly positively correlated with dry matter accumulation of *D. nobile* (*P* < 0.05) and significantly negatively correlated with Fe (*P* < 0.05); and the biomass of bryophytes was significantly negatively correlated with Mg, Fe and Mn (*P* < 0.05).

### Comprehensive evaluation of yield, quality and mineral element content of *D. nobile* from 35 quadrats

According to the results of the correlation analysis, the correlation was weak among the biomass, quality and mineral content of *D. nobile* associated with different species of bryophytes. The nonsignificant correlation between the indicators may lead to differences between the results of the comprehensive evaluation of the yield and quality of *D. nobile* in the 35 quadrats using multiple indicators and single indicators. Therefore, to clarify the influence of different indexes on *D. nobile*, PCA and membership function were used to comprehensively evaluate the biomass, quality and mineral content of *D. nobile* and to explore the influence of different species of associated bryophytes on the yield and quality of *D. nobile*. The dry matter accumulation of 1-, 2- and 3-year-old stems of *D. nobile* in this experiment varied greatly among the 35 quadrats. Therefore, the biomass of each quadrat was calculated as the total dry matter accumulation of 1-, 2- and 3-year-old stems. Among the quality indicators, the coefficient of variation of leachate content was 5.46%, which was smaller, while the coefficient of variation of dendrobine content was 21.08%, which was larger, so dendrobine content was used as an indicator for quality evaluation of the samples from the 35 quadrats. However, the coefficient of variation for the content of all 11 mineral elements was large. Therefore, all elements were used as evaluation indicators. We ranked each quadrat according to its C (standardized scores of principal components with eigenvalues greater than 1 in PCA) and D (comprehensive membership function value) values, and the larger the value was, the higher the evaluation. In addition, the single-indicator evaluations of dry matter and quality were ranked according to the size of their measured values.

The scores and ranking results, single indicator evaluation and multi-indicator comprehensive evaluation of dry matter, quality and mineral element content of *D. nobile* from 35 quadrats are shown in Table 5. There were four common quadrats in the top 10 of the PCA and membership function evaluation for multiple indicators (dry matter, quality, and mineral elements), namely, D5, D15, D17, and D21, and all were ranked relatively high. The similarity between the PCA and the membership function evaluation of the mineral elements and the evaluation results of their corresponding multi-indicators were high. There were seven identical quadrats in the top 10 of the two principal component analyses (D4, D5, D8, D15, D17, D21, and D30), and seven identical quadrats in the top 10 of the two membership function evaluations (D5, D6, D15, D17, D20, D31, and D32) but differences were observed in quadrat identity. This indicates that there were large differences among different evaluation methods for the comprehensive evaluation of yield and quality of *D. nobile*. In the dry matter evaluation and comprehensive PCA and membership function evaluation of multiple indicators, there were 2 and 3 common quadrats among the top 10, respectively, with D4 and D5 in the PCA and D5, D10 and D20 in the membership function evaluation. In the dendrobine content evaluation and comprehensive PCA and membership function evaluation of multiple indicators, there were 7 and 4 common quadrats the top 10, respectively, with D1, D5, D8, D9, D15, D21, and D30 in the PCA and D5, D10, D15, and D21 in the membership function evaluation. In the dry matter evaluation and the comprehensive PCA and membership function evaluation of mineral elements, there were 3 and 2 common quadrats in the top 10, respectively, with D4, D5, and D23 in the PCA and D5 and D20 in the membership function. In the dendrobine content evaluation and comprehensive PCA and membership function evaluation of mineral elements, there were 5 and 3 common quadrat in the top 10, respectively, with D5, D8, D15, D21 and D30 in the PCA and D5, D15 and D21 in the membership function evaluation.

**Table 5:** Top 10 ranked quadrats in the comprehensive evaluation of dry matter, dendrobine and mineral elements in *D. nobile*

Ranking	Dry matter		Dendrobine		Multiple mineral elements				Multiple indicators (dry matter, dendrobine and mineral elements)			
					PCA		Membership function		PCA		Membership function	
1	D12	1.754	D21	0.81	D15	1.159	D5	0.554	D5	1.411	D5	0.668
2	D5	1.753	D9	0.68	D5	0.943	D17	0.526	D21	1.006	D17	0.569
3	D23	1.547	D5	0.68	D17	0.546	D32	0.509	D15	0.884	D21	0.555
4	D22	1.466	D16	0.66	D19	0.533	D21	0.461	D17	0.747	D32	0.531
5	D18	1.462	D8	0.65	D23	0.521	D6	0.430	D8	0.357	D20	0.484
6	D20	1.444	D15	0.62	D8	0.433	D20	0.426	D9	0.295	D15	0.466
7	D13	1.36	D1	0.61	D21	0.417	D31	0.407	D4	0.256	D6	0.454
8	D14	1.348	D30	0.59	D30	0.396	D15	0.396	D30	0.253	D10	0.445
9	D4	1.316	D10	0.57	D29	0.355	D24	0.391	D1	0.217	D31	0.439
10	D10	1.298	D35	0.55	D4	0.353	D29	0.388	D23	0.185	D11	0.439

In summary, the similarity of the results obtained was not high between the PCA and membership function evaluation, with only 40% of the same quadrats being among the top 10. The order of similarity among the dry matter, dendrobine, and mineral element results when evaluated individually and the comprehensive results of multiple indicators was mineral elements > dendrobine > dry matter. This indicates that mineral elements are given more weight than dry matter and dendrobine when performing a comprehensive evaluation.

The top 10 quadrats were obtained according to each evaluation method, and then the frequency of each bryophyte and its coverage in these 10 quadrats were determined. We identified the bryophyte species with a frequency of occurrence greater than 5 as the preliminarily identified dominant bryophytes associated with *D. nobile*, and the statistical results are shown in Table 6. As shown in the table, the preliminary identification of dominant associated bryophytes screened by all four evaluation methods included *L. juniperoideum* and *T. microphylla*. Among these methods, *T. microphylla* (9 times) and *L. juniperoideum* (10 times), had the strongest correlation with dry matter accumulation as they were significantly more frequent in this evaluation than in the evaluation methods using dendrobine, mineral elements, and multiple indicators. By comparing the coverage of dominant bryophytes in each preliminary identification, the results showed that the coverage of *T. microphylla* in the evaluation using dry matter and dendrobine was 67.22% and 67.50%, respectively, which was significantly higher than that in the comprehensive evaluation using mineral elements and multiple indicators, but the difference in the coverage of *L. juniperoideum* was not significant among different evaluation methods. In summary, *T. microphylla* and *L. juniperoideum* were identified as the dominant bryophytes associated with *D. nobile*.

**Table 6:** Frequency and coverage of *D. nobile*-associated bryophytes preliminarily identified as dominant (frequency > 5)

Dry matter		Dendrobine		Mineral elements		Comprehensive evaluation of dry matter, dendrobine and mineral elements	
Moss species	Frequency (coverage/%)	Moss species	Frequency (coverage/%)	Moss species	Frequency (coverage/%)	Moss species	Frequency (coverage/%)
*T. microphylla*	9 (67.22)	*T. microphylla*	6 (67.50)	*T. microphylla*	6 (57.50)	*T. microphylla*	6 (60.00)
*L. juniperoideum*	10 (15.80)	*L. juniperoideum*	7 (19.57)	*L. juniperoideum*	6 (13.67)	*L. juniperoideum*	6 (16.00)
				*R. cuspidigerum*	5 (16.20)	*B. glaciale*	5 (28.20)
				*B. glaciale*	5 (28.20)	*T. kanedae*	5 (8.60)
				*T. kanedae*	5 (8.60)		

## Discussion

### Effects of microenvironmental differences in Danxia stone on bryophyte species composition

The distribution of different species is related to biotic and abiotic environmental characteristics [23, 24], including forest structure, substrate, microorganisms and climatic conditions [25, 26]. The occurrence and persistence of bryophytes depends largely on the availability of nutrients, water and shade [27–29]. Human activities (e.g., deforestation, cultivation, agriculture, urban expansion) are one of the main drivers of biodiversity change [30]. In the study area, the technique of cultivating *D. nobile* attached to Danxia stone was used based on the plant’s unique growth environment requirements, which includes cleaning of the Danxia stone and its surroundings prior to planting. First, a brush is used to clean the surface of the Danxia stone of soil, deadfall and bryophytes to ensure that the *D. nobile* roots can attach smoothly. Second, if the tree density is high, some of the trees around the Danxia stone must be cut down to ensure that it receives sufficient sunlight. If there are no trees around the Danxia stone, to avoid direct sunlight in the summer, a shade net above is placed above the Danxia stone. Finally, to cope with extreme drought conditions, a sprinkler system is installed in the production area using pipes for proper irrigation. Under the interference of these anthropogenic measures, the microenvironment (temperature, light intensity, sunshine hours, moisture, nutrients) of the surface layer of the Danxia stone is altered, which provides the possibility for colonising bryophytes survive or undergo turnover, e.g., *T. microphylla* and *L. juniperoideum* can only survive in the Danxia stone, which has a high degree of shading and moisture content. In addition, the successful colonisation of *D. nobile* provided a more favourable environment for the growth of bryophytes. The root system of *D. nobile* is attached to the rock surface, and its roots are made up of a rare spongy tissue with excellent aeration, adsorption and water retention [31], which improves the microenvironment of the Danxia stone surface layer, making it more conducive to bryophyte attachment. Additionally, *D. nobile* and associate bryophytes interact with each other to improve each other's adaptive ability to grow on Danxia stone. In this study, we found that differences in the microenvironments (e.g., moisture content, light intensity and temperature) of Danxia stone may be the determining factor for the different species compositions of bryophytes. The moisture content of the 35 Danxia stones ranged from 0.91–10.47%, with a coefficient of variation of 59.61%, and it was significantly and positively correlated with the moisture content of the bryophytes (*P* < 0.05). In addition, we found significant differences in shade, light intensity, hours of sunshine and surface temperature among the Danxia stones through field observations. The bryophyte species (3–9 species), coverage (1–95%), and biomass accumulation (0.38–5.60 g/clump) of the 35 Danxia stones also varied considerably, and the moisture content of Danxia stones was significantly and positively correlated with the moisture content and biomass of bryophytes (*P* < 0.05). Therefore, we suggest that the microenvironment of Danxia stone plays an important role in bryophyte species composition.

### Effect of differences in bryophytes species on the growth of *D. nobile*

Although research on how bryophytes influence vascular plants has attracted increasing attention during the last decade, much is still unknown about the driving processes behind these effects [12]. The aim of this study was to understand the effects of different species of associated bryophytes on the yield and quality of *D. nobile* and to identify the dominant bryophyte species driving these factors. The results showed that the coefficients of variation of biomass, dendrobine content and mineral content of *D. nobile* were higher in different species of associated bryophytes, ranging from 40.13%, 21.08%, and 11.33–57.96%, respectively. This indicates a strong correlation between different species of associated bryophytes and the growth of *D. nobile*. This finding is consistent with previous findings that the presence of bryophytes can increase the aboveground biomass and height of plants [32, 33]. Although bryophytes are widespread on rocks, the bryophyte layer is highly variable due to differences in their species composition [34, 35]. Bryophytes have many different morphological and physiological characteristics that can have different effects on plants. The reason for this is that the water retention capacity of different bryophytes varies considerably [36, 37], determining the severity of drought exposure of the bryophyte layer, which shows the same effect on the ability of associated plants in resisting drought [38, 39]. This was also confirmed by the significant positive correlation (*P* < 0.05) between the moisture content of the bryophytes and the biomass accumulation of *D. nobile* in the present study, demonstrating that the water-holding properties of bryophytes play an important role in the biomass accumulation of *D. nobile*. However, it is worth noting that the correlation between the biomass of bryophytes and the yield and quality of *D. nobile* was not significant, indicating that the thickness and biomass of bryophytes are not the key factors determining the yield and quality of *D. nobile* during its growth. In addition, due to differences in morphological and physiological characteristics, bryophyte species also differ in competition for space [7, 40] and in their ability to intercept and retain nitrogen [12] and take up phosphate, among other factors [41], which can have different effects on the growth of *D. nobile*, but quantifying these effects requires further research.

In this study, *T. microphylla* and *L. juniperoideum* were identified as the dominant associated bryophytes of *D. nobile*. Both bryophytes showed morphological characteristics of small plant size, erect stems, and dense clumps [42–44] and had strong water-holding properties. The remaining bryophytes performed poorly in terms of water-holding properties, e.g., *Hypnum* species had long, creeping stems that were more branched and loosely interwoven into sheets. The stems of *Fissidens* are single, rarely branched, and sparsely arranged among the plant bodies. Although small and densely clumped, *Bryum* species prefer to grow on slightly drier rocks [44]. As a result, the morphological characteristics and habits of these bryophytes lead to poor water-holding properties. In addition, by observing the growth of the *D. nobile* root system with bryophytes, we found that *T. microphylla* and *L. juniperoideum*, due to their dense clumping and tight arrangement, could better wrap the root system of *D. nobile* and keep it moist even under long-term drought conditions, which ensured the normal growth of *D. nobile* to the maximum extent.

In summary, we make the following preliminary generalisations about the formation of the microenvironments of Danxia stone, *D. nobile* and bryophyte. The differences in the microenvironments (e.g., shade, direction, water and humidity conditions) in which the Danxia stones are located, as well as the intervention of anthropogenic measures during the planting and management of *D. nobile*, led to changes in the microenvironments of the Danxia stone. That is, there were subtle differences in environmental factors such as moisture, nutrients, light intensity, sunshine hours and temperature in the surface layer of the Danxia stone, which provide suitable environments for the growth of different species of bryophytes. Additionally, after the successful colonisation of bryophytes and *D. nobile*, the three components interacted with each other and gradually formed a stable mini-ecosystem that continuously influenced the growth of *D. nobile*.

The present study focuses on the important role of moisture in the microenvironment of *D. nobile* with respect to the experimental results, but the effects of the remaining microenvironmental factors, such as light, nutrients, temperature, and microbial activity, cannot be ignored. Therefore, rational experiments should be designed for these microenvironmental factors in subsequent studies to comprehensively understand the effects of associated bryophytes on the growth of *D. nobile*. To explore the effects of these factors on the growth of *D. nobile* and to study the interactions between them to more systematically clarify the formation mechanism of the microenvironment of Danxia stone, *D. nobile* and bryophytes. In addition, in this study, two associated bryophytes, *T. microphylla* and *L. juniperoideum*, were found to be associated with the yield and quality of *D. nobile*. However, we have not been able to fully reveal the mechanism of action of these two bryophytes on the yield and quality of *D. nobile*. This is an important direction for our team's future research, and we intent to set up a rational experimental program for its study.

## Conclusion

In summary, there is a rich variety of *D. nobile*-associated bryophytes. A total of 15 families, 24 genera and 31 species of bryophytes were identified in the study area, including 13 families, 22 genera and 29 species of mosses and 2 families, 2 genera and 2 species of liverworts, and mosses predominated in the associations with *D. nobile*. Mixed-species associations of 5–6 bryophyte species were more common. Different associated bryophytes had a greater effect on the dry matter accumulation, quality and mineral content of *D. nobile*. *T. microphylla* and *L. juniperoideum* were identified as dominant associated bryophytes based on the PCA and the membership function, and these two bryophytes were associated with *D. nobile* through mixed growth.

## Materials and methods

### Study location

**Fig. 10:**
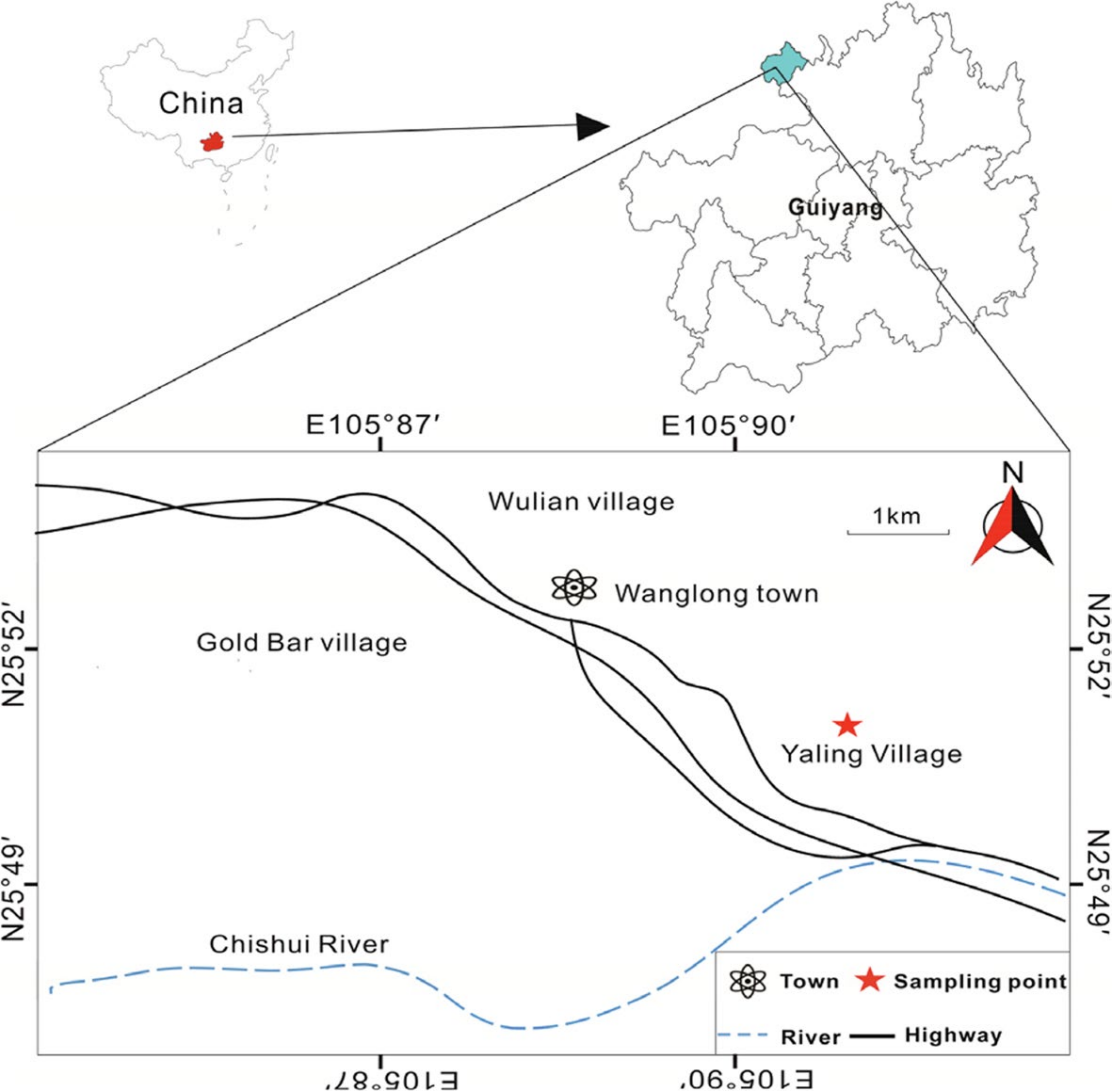
Geographical location of sampling points

The test site selected for this study was Yaling Village, Wanglong Town, Chishui City, Guizhou Province, where *D. nobile* was cultivated by a centralised and continuous planting method in an arboreal understorey. Wanglong Town (105°90′N, 28°52′E) is located in the central part of Chishui City, one of the main production areas of Chishui *D. nobile*. The *D. nobile* planting base in Yuling Village (105°91′E, 28°51′N), at an altitude of 500 m, has the largest concentrated and continuous planting area of *D. nobile* in Chishui City (more than 134 hectares). Wanglong Town is located in the river valley semi-alpine hills, and the terrain is high in the southeast and low in the northwest, belonging to the subtropical monsoon climate, with an altitude of 228–1,256 m. According to the Chishui Meteorological Bureau, the average annual rainfall in Wanglong Town from 2012 to 2022 is 785.11–1,658.37 mm, and the average annual temperature is 17.37–18.79℃. The exact location of the study area is shown in Fig. 10.

### Test materials

*D. nobile*: It was identified as *D. nobile* of the genus *Dendrobium*, family Orchidaceae, by Professor Zhi Zhao of the College of Agriculture, Guizhou University. It had been planted for 3 years.

Danxia stone: Sandstone rock properties.

Bryophyte: Bryophyte associated only with the root system of *D. nobile*.

### Main instrumentation

Plasma emission spectrometry (ICP‒OES Optima 8000), fully automated Kjeldahl nitrogen determination (HGK-55), and gas chromatography (Agilent 7890A) were performed.

### Sample collection

In this experiment, bryophyte specimens were collected twice in the study area, in September 2021 and May 2022. Only the bryophytes associated with the *D. nobile* root system on Danxia stone were collected so that the collected specimens would represent the status of bryophytes associated with *D. nobile* in the region. A total of 163 specimens were collected and taken to the laboratory for drying and identification to the species level. Additionally, 35 Danxia stones were randomly selected in the study area, and the selection criterion was that the area of one face of the Danxia stone was larger than 1.5 m × 1.5 m, and the face was set as one quadrat, for a total of 35 quadrats. To ensure that the selected quadrat was representative of *D. nobile* growth and bryophyte distribution in the study area, Danxia stones with differences in direction, shade and slope were selected. The details of the 35 quadrats are shown in Table 7. We collected samples of *D. nobile*, bryophyte and Danxia stone within quadrats and chose to collect samples on an overcast day on 5 May 2022, after three consecutive sunny days, to minimise experimental error. The specific sampling steps were as follows:

**Table 7:** Basic information on the 35 Danxia stones sampled

Number	Direction	Slope/°	Moisture content/%	Number	Direction	Slope/°	Moisture content/%	Number	Direction	Slope/°	Moisture content/%
D1	Northeast	90	2.57	D13	West	90	4.34	D25	Southwest	90	7.89
D2	West	90	1.01	D14	North	90	6.64	D26	Northeast	90	1.77
D3	Northeast	90	6.16	D15	East	90	1.45	D27	Northeast	90	6.37
D4	Northwest	90	7.28	D16	Northeast	90	10.47	D28	West	90	2.77
D5	North	90	6.11	D17	West	45	1.21	D29	Southeast	90	1.07
D6	Towards the top	180	1.51	D18	North	90	7.13	D30	West	90	1.67
D7	Towards the top	180	1.35	D19	East	90	1.10	D31	Northeast	90	3.55
D8	West	80	6.71	D20	North	30	1.98	D32	Northeast	90	4.76
D9	East	80	6.48	D21	North	60	6.08	D33	Northwest	90	6.07
D10	Northwest	90	3.54	D22	Northeast	80	6.94	D34	North	90	2.61
D11	North	90	7.48	D23	West	90	0.91	D35	West	90	8.68
D12	North	30	6.95	D24	Northwest	90	8.35

- ① *D. nobile* sample collection: In each quadrat, 15 clumps of *D. nobile* with similar growth were selected, and one 1-, 2- and 3-year-old stems were cut off from each clump of *D. nobile* with scissors. The leaves were removed, and the 15 stems were mixed into one composite sample, which was transported to the laboratory for the determination of dry matter accumulation, quality, and mineral element contents of *D. nobile* stems. ② Bryophyte sample collection: Samples of all species of bryophytes within the quadrat were collected into self-sealing bags and labelled, and the cover of each bryophyte was counted; 182 samples were collected in total. Next, three clumps of *D. nobile* were randomly selected from among 15 clumps of *D. nobile* and an area of 10 cm × 10 cm centred around the base of the stems was delimited. All bryophytes within the delineated area were scraped with a knife, the roots and other debris were removed, and the collected bryophytes were packed in a self-sealing bag. A total of 105 samples were collected and transported to the laboratory to determine the fresh and dry weights, moisture content and biomass of the bryophytes. ③ Danxia stone sample collection: Three clumps of *D. nobile* were selected from each quadrat, and Danxia stone samples were collected from the delineated area after the bryophyte samples were collected. Approximately 0.5 cm of the surface layer of Danxia stone was chiselled with a geological hammer to obtain lumpy or powdery samples of Danxia stone. Roots and other debris were removed, and the obtained samples of Danxia stone were packed in self-sealing bags. A total of 105 samples of Danxia stone were collected, and their fresh and dry weights and moisture content were determined (Table 7).

### Measurements

#### Determination of biomass, quality and mineral content of *D. nobile*

##### Biomass determination

The dry weight of 15 stems per sample was determined as follows: first, the fresh weight (g/branch) of the 15 stems was determined in the natural water content state of the plant, and second, the total fresh weight of the 15 stems was determined. Subsequently, the stems were cut into small sections and dried in an oven at 60 °C to a constant weight. The dry weight was determined, and the drying rate of the stems was calculated. Finally, the dry weight of a single stem was calculated based on the drying rate (g/branch). Drying rate formula:

1 $$\mathrm{Drying\;rate}\;\left(\%\right)=\frac{\mathrm{Fresh}\;\mathrm{weight}\;\mathrm{of}\;Dendrobium\;nobile}{\mathrm{dry}\;\mathrm{weight}\;\mathrm{of}\;Dendrobium\;nobile}$$

##### Quality determination

Measurement of leachate content: Determination of ethanol leachate was carried out according to the hot leaching method under the method for determination of alcohol-soluble leachate in Part IV of the 2020 edition of the Chinese Pharmacopoeia (General rule 2201) [45].

Determination of dendrobine content: The content of dendrobine was determined by gas chromatography (General rule 0521) with reference to the 2020 edition of the Chinese Pharmacopoeia [46]. Dendrobine control solution was prepared at a mass concentration of 52.5 μg·mL^−1^, and 1 μl was aspirated and injected into a gas chromatograph to record the chromatogram of dendrobine. The chromatographic column was a DB-1 capillary column with a programmed temperature increase: the initial temperature was 80 ℃, and then the temperature was increased to 250 ℃ at a rate of 10 ℃ per minute and held for 5 min. The temperature of the sample inlet and the FID detector was 250 ℃, the carrier gas was nitrogen, the split ratio was 5:1, the flow rate of hydrogen was 30 mL·min^−1^, the flow rate of air was 300 mL·min^−1^, and the flow rate of the exhaust gas was 30 mL·min^−1^. In addition, the theoretical plate number was not less than 10,000. The standard curve was plotted using the concentration as the horizontal coordinate and the peak area ratio (concentration of each reference standard(s) required/peak area of the internal standard) as the vertical coordinate. The standard curve equation, *y* = 0.102*x*–0.06 (*R*^2^ = 0.9997), indicates that dendrobine has good linearity in the range of 1.0–20.0 μg·mL^−1^.

##### Mineral element content determination

Elemental N content was determined by using a Kjeldahl nitrogen apparatus, in accordance with the 2020 edition of the Chinese Pharmacopoeia (General rule 0731) for protein content determination – Kjeldahl method for nitrogen determination [45]. The elemental contents of K, Ca, P, Mg, Na, Fe, Cu, Zn, Mn and B were determined by plasma emission spectrometry (ICP‒OES), and reference standards for each element were provided by the China Nonferrous Metals and Electronic Materials Analysis and Testing Centre.

#### Bryophyte species identification and frequency, cover, biomass and moisture content determination

Species identification: The collected specimens were identified to species by Professor Yuanxin Xiong and Associate Professor Wei Cao from the School of Life Sciences, Guizhou University.

##### Frequency, coverage and moisture content calculations

Frequency: the number of occurrences of each bryophyte in all quadrats; Frequency (%): the number of times a bryophyte occurs as a proportion of the number of all quadrats; Cover (%): estimated using the grid method, i.e., the proportion of the growth area of one type of bryophyte within the quadrat to the area of the Danxia stone, expressed as a percentage; Moisture content (%): three clumps of *D. nobile* were randomly selected from each quadrat, an area of 10 cm × 10 cm was delineated with the base of *D. nobile* stems as the centre, and all the bryophytes within the area were scraped with a knife to remove the root system and other debris. The bryophytes obtained were packed in self-sealing bags and taken to the laboratory to be weighed fresh. Subsequently, the bryophytes were dried to obtain a dry weight and calculate their moisture content. The frequency and moisture content were calculated by the following formulas:

2 $$\mathrm{Frequency}\;\left(\%\right)=\frac{\mathrm{Frequency}\;\mathrm{of}\;\mathrm{occurrence}\;\mathrm{of}\;\mathrm{bryophyte}}{\mathrm{Total}\;\mathrm{number}\;\mathrm{of}\;\mathrm{quadrats}}$$

3 $$\mathrm{Moisture\;content}=\frac{\mathrm{Moisture}\;\mathrm{weight}}{\mathrm{Dry}\;\mathrm{weight}\;\mathrm{of}\;\mathrm{bryophyte}}$$

#### Moisture content of Danxia stone

Three clumps of *D. nobile* selected based on the bryophyte moisture content determination were delineated in a 10 cm × 10 cm area centred on the base of *D. nobile* stems. The surface layer of the Danxia stone was chiselled with a geological hammer to approximately 0.5 cm from the surface to obtain lumpy and powdered samples, and roots and other debris were removed. The obtained Danxia stone samples were packed in self-sealing bags and taken to the laboratory to be weighed fresh. Subsequently, the samples were dried to obtain a dry weight and calculate their moisture content. The moisture content was calculated by the following formula:

4 $$\mathrm{Moisture\;content}\;\left(\%\right)=\frac{\mathrm{Moisture}\;\mathrm{weight}}{\mathrm{Dry}\;\mathrm{weight}\;\mathrm{of}\;\mathrm{Danxia\;stone}}$$

### Data processing and statistical analysis

SPSS 25.0 and Excel 2019 were applied to statistically analyse the data, and Origin 9.8 with CoreIDRAW 2020 software was used for graphing. We used principal component analysis (PCA) and a membership function to conduct a comprehensive evaluation.

PCA: Data were standardised for each indicator using SPSS 25.0 software, and factors with eigenvalues greater than 1 were extracted as principal components. The coefficients of each principal component composite indicator were obtained by PCA, and the corresponding product was obtained to calculate the score (C-value) of each quadrat composite indicator [47].

Membership function evaluation: The value of the membership function *U (x)* for each quadrat was calculated according to Eq. (5), the weight of each indicator was calculated according to Eq. (6), and the value of the composite score for each sample (D-value) was derived using Eq. (7) [48, 49].

5 $${U}_{Xij} = \frac{X-Xmin}{\left(Xmax -Xmin\right)} ; \left(i =1, 2, 3,\dots ,\mathrm{ n}\right)$$

where *X* is the measured value, *Xmax* is the maximum value and *Xmin* is the minimum value.

6 $${W}_{i} = \frac{Vi}{Vn}$$

where *Wi* denotes the weight of the i-th indicator extracted, expressed as a percentage, *Vi* is the coefficient of variation of indicator *i* (*i* = 1, 2, 3,…, n), and *Vn* is the sum of the n coefficients of variation sought.

7 $$\mathrm{Comprehensive}\;\mathrm{membership}\;\mathrm{function}\;\mathrm{value}\;\left(\mathrm D-\mathrm{value}\right)=\frac{\sum\left[U\left(Xij\right)\times Wi\right]}{Wn}$$

where *Wn* represents the sum of the indicator percentages.

## Data Availability

The datasets used and/or analysed during the current study are available from the corresponding authors upon reasonable request.

## Abbreviations

N: Nitrogen
K: Potassium
Ca: Calcium
P: Phosphorus
Mg: Magnesium
Na: Sodium
Fe: Iron
Cu: Copper
Zn: Zinc
Mn: Manganese
B: Boron
C-value: Scores from principal component analysis
D-value: Score of the membership function
PCA: Principal component analysis
